# Identification of ARNT-regulated BIRC3 as the target factor in cadmium renal toxicity

**DOI:** 10.1038/s41598-017-17494-9

**Published:** 2017-12-11

**Authors:** Jin-Yong Lee, Maki Tokumoto, Gi-Wook Hwang, Moo-Yeol Lee, Masahiko Satoh

**Affiliations:** 1 Laboratory of Pharmaceutical Health Sciences, School of Pharmacy, Aichi Gakuin University, 1–100 Kusumoto-cho, Chikusa-ku, Nagoya, Aichi 464–8650 Japan; 2 Laboratory of Molecular and Biochemical Toxicology, Graduate School of Pharmaceutical Sciences, Tohoku University, Sendai, 980-8578 Japan; 3 College of Pharmacy, Dongguk University, Goyang, Gyeonggi-do, 410-820 Republic of Korea

## Abstract

Cadmium (Cd) is an environmental contaminant that exhibits renal toxicity. The target transcription factors involved in Cd renal toxicity are still unknown. In this study, we demonstrated that Cd decreased the activity of the ARNT transcription factor, and knockdown of *ARNT* significantly decreased the viability of human proximal tubular HK-2 cells. Microarray analysis in *ARNT* knockdown cells revealed a decrease in the expression of a number of genes, including a known apoptosis inhibitor, BIRC3, whose gene and protein expression level was also decreased by Cd treatment. Although the BIRC family consists of 8 members, Cd suppressed only *BIRC3* gene expression. BIRC3 is known to suppress apoptosis through the inhibition effect on caspase-3. Knockdown of *BIRC3* by siRNA as well as Cd treatment increased the level of active caspase-3. Moreover, knockdown of *BIRC3* not only triggered cell toxicity and apoptosis but also strengthened Cd toxicity in HK-2 cells. Meanwhile, the activation of caspase-3 by suppression of *BIRC3* gene expression was mostly specific to Cd and to proximal tubular cells. These results suggest that Cd induces apoptosis through the inhibition of ARNT-regulated BIRC3 in human proximal tubular cells.

## Introduction

Cadmium (Cd) is an environmental contaminant that induces toxic effects in various tissues including the kidney^1–3^. Cd accumulates in many organs, particularly in the kidney, because of its long biological half-life (10–30 years)^1^. Chronic Cd exposure arises mainly from dietary source and cigarette smoking and can cause nephrotoxicity, osteomalacia, teratogenicity and reproductive dysfunction^4,5^. In chronic dietary Cd exposure, the kidney is the target organ. Proximal tubular cell damage is characterized as Cd-induced renal damage^1^. Renal tubular cells take up Cd as a detoxified form bound to metallothionein (MT), and Cd is released from the bound form after lysosomal metabolism^2^. Although unbound Cd stimulates MT production in the kidney and then binds to MT, the toxic effects occur when unbound Cd accumulates in renal tubular cells^2,6^. At the molecular level, Cd induces apoptosis and also has adverse effects on cellular proliferation, cell signaling, and DNA repair in various cell lines^7–10^. Cd induces apoptotic cell death through endoplasmic reticulum (ER)-mediated, mitochondrial-mediated and p53-mediated pathways^8^. Cd impairs cell survival and proliferation through an increase in the phosphorylation of c-Jun NH2-terminal kinase (JNK) and p38 MAP kinase^10^. Cd inhibits the initial steps of base excision repair system and suppresses the capacity of the nucleotide excision system through interference with various enzymes^9^. Although Cd has been reported to have adverse effect on cellular functions, the precise mechanism of Cd-induced proximal tubular cell toxicity remains unclear.

To elucidate the precise mechanism of Cd-induced renal toxicity, we previously used the DNA microarray method to screen genes whose expression is changed by Cd treatment in mouse kidney and cultured cells, including proximal tubular cells^11–13^. Among the genes whose expression is downregulated by Cd treatment, the genes coding for proteins involved in the ubiquitin-proteasome system (UPS) are associated with Cd-induced renal toxicity^14,15^. Previous studies demonstrated an association between increased gene expression of apoptotic factors and Cd-induced apoptosis^8,14,15^. Other research groups revealed that the disruption of gene expression is involved in Cd-induced renal toxicity^16–18^. These results strongly imply that Cd exerts cytotoxicity through the disruption of gene expression.

The transcriptional regulation of eukaryotic genes involves the organized assembly of multi-protein complexes on promoter regions^19,20^. However, the upstream pathways that regulate gene transcription are controlled by specific regulatory mechanisms; furthermore, not all genes are induced at the same time and with the same duration. Some genes, such as those responsible for correct protein folding, are immediately induced for transcription within minutes; whereas others, such as those involved in DNA damage repair and cell metabolism, are slowly responded to upstream inductions signaling, within hours^21^. Once activated, transcription factors bind to gene regulatory elements (*cis*-elements), and through interactions with co-factors of the transcription machinery, promote access to DNA and facilitate the recruitment of the RNA polymerase enzymes to the transcriptional start site^19,22^.

Previous studies have shown that Cd exposure enhances the activities of transcription factors that induce cellular protection pathways. For instance, metal response element (MRE)-binding transcription factor-1 (MTF-1) is activated by Cd and induces transcription of MTs^23–25^. The Nrf2 transcription factor is also reported to play a role in Cd exposure-mediated gene expression of MT^26^. Although several transcription factors involved in MT expression have been identified, the transcription factors that control the expression of genes associated with Cd renal toxicity are poorly understood.

In this study, we screened transcription factors whose activities were changed by Cd treatment in HK-2 human proximal tubular cells. Among the transcription factors affected by Cd, we investigated the Cd-targeted transcription factors essential for Cd-induced renal toxicity. Furthermore, we examined the downstream factors of Cd-targeted transcription factors that are involved in Cd-induced renal toxicity.

## Results

### Identification of the transcription factors with altered binding activity in response to Cd treatment in HK-2 cells

**Figure 1:**
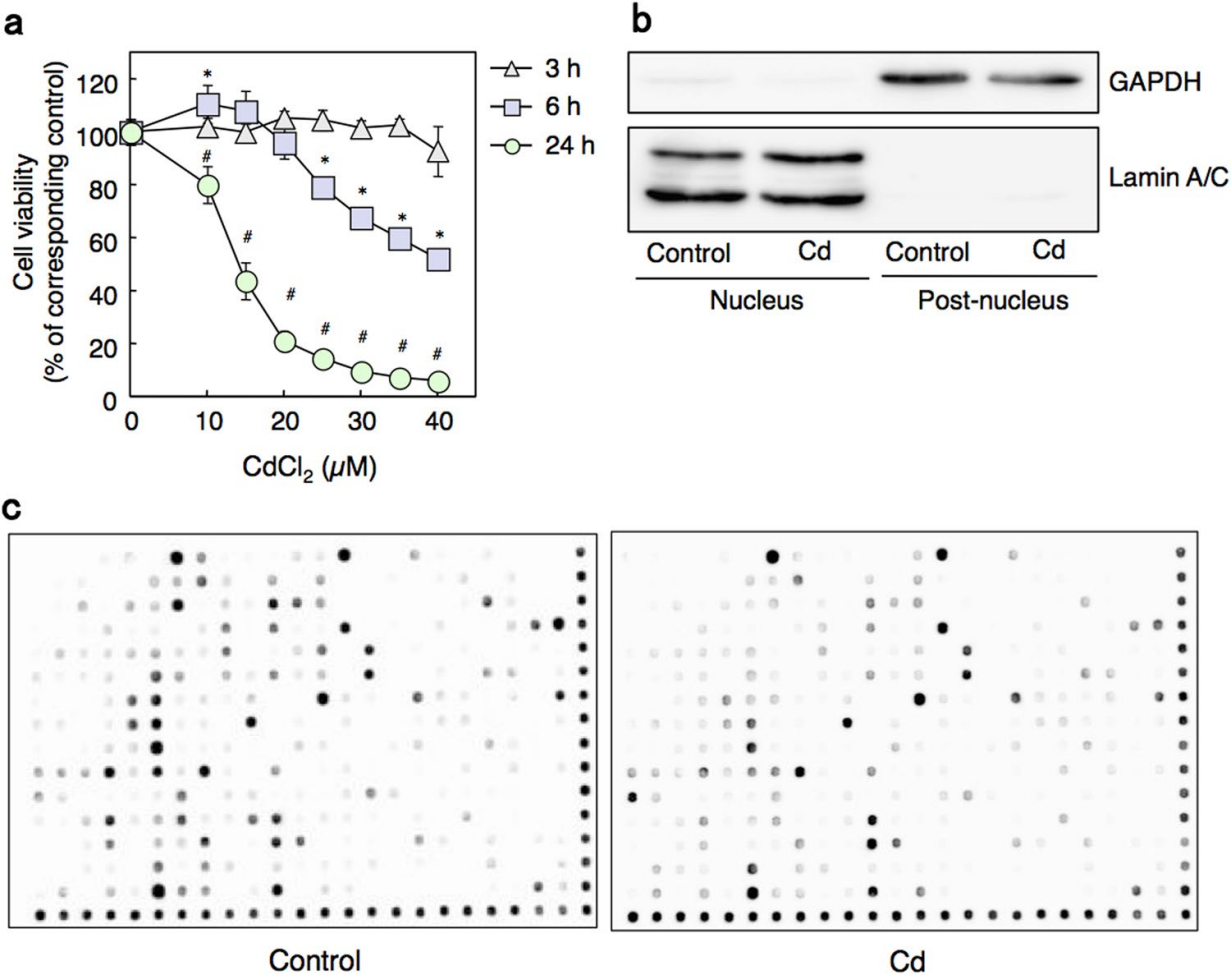
Protein/DNA binding array with nuclear extracts from HK-2 cells treated with Cd. (**a**) Cell viability of HK-2 cells after treatment with Cd for 3, 6 or 24 h using the MTT assay. Values are the means ± S.D. (*n* = 4 or 5). *Significantly different from the control group of 6 h treated group, *P* < 0.05. ^#^Significantly different from the control group of 24 h treated group, *P* < 0.05. The absence of an error bar indicates that the S.D. was within the area of the symbol. (**b**) Western blot confirmation of nuclear extracts from control and Cd-treated HK-2 cells. HK-2 cells treated as indicated were separated into nuclear and post-nuclear fractions and western blot analysis was conducted to confirm the fractionation process. Lamin A/C, nuclear marker; GAPDH, cytosolic marker. The blots were run under the same experimental conditions and cropped from same membrane. Uncropped images are provided in Supplementary Fig. 2a. (**c**) DNA-binding activity of transcription factors in HK-2 cells treated with Cd using the Combo Protein/DNA Array. The nuclear extracts from control cells or cells treated with 40 µM Cd for 3 h were used for protein/DNA binding array. Spots along the right side and bottom of the membranes are biotinylated DNA for normalization.

To investigate Cd-induced cytotoxicity in HK-2 cells, we first examined cell viability of HK-2 cells treated with various concentrations of Cd using the MTT [3-(4,5-Dimethyl-2-thiazolyl)-2,5-diphenyl-2*H*-tetrazolium bromide] assay (Fig. 1a). HK-2 cells treated with 40 µM Cd for 6 h showed 50% cell toxicity and exhibited less than 10% cell viability at 24 h; however, at 3 h, 40 µM Cd treatment did not induce cytotoxicity. Therefore, we selected these conditions, in which cells remain viable even with Cd treatment (40 µM for 3 h), to examine Cd activation-changed transcription factors using protein/DNA binding arrays in nuclear extracts^27^. Figure 1b shows confirmation and purity of our nuclear extraction process, in which the nuclear marker Lamin A/C is detected in nuclear fractions from both control and Cd-treated cells. Nuclear extracts from control and Cd-treated cells were then incubated with a mixture of 345 biotin-labeled DNA probes corresponding to transcription factor response elements (*cis*-elements). The quantity of *cis*-elements was determined using the protein/DNA binding array membrane on which elements that would hybridize with any *cis*-elements present were pre-spotted. Cd treatment affected the binding of several transcription factors to *cis*-elements compared with the control group (Fig. 1c). Overall, Cd increased the binding activities of 20 transcription factors by more than 2-fold (Table 1). Our results showed that binding activities of both p53 and MTF-1 transcription factors were increased in HK-2 cells in response to Cd treatment. We previously demonstrated that p53 protein level was dramatically increased by Cd treatment *in vivo* and *in vitro* ^14,15^. Moreover, another study demonstrated that the expression of apoptosis genes regulated by p53 is increased by Cd treatment in HK-2 cells^14^. A previous study showed that MTF-1 is induced to bind the MRE by Cd^23–25^. Our results are consistent with these findings. Cd also decreased the binding activities of 28 transcription factors, including PPAR (peroxisome proliferator activated receptor alpha) and Sp-1, by less than 0.5-fold (Table 2). Consistent with our results, several previous studies reported that PPAR and Sp-1 activities were decreased by Cd^28,29^. These studies help validate our findings and indicate our results may provide valuable information about the transcription factors involved in Cd toxicity or protection against Cd toxicity.

**Table 1:** Transcription factors with increased binding activities in response to Cd treatment. HK-2 cells were treated with 40 µM Cd in serum-free culture medium for 3 h. The cells were separated into nuclear and post-nuclear fractions. The protein/DNA binding array was performed using the Combo Protein/DNA Array. Spot density was evaluated using ImageQuantTL software. Transcription factors whose activities were increased 2-fold or more are listed.

Name	Description	Ratio
p53	Tumor protein p53	95.50
SIF-2	SI promoter 2	22.00
myc/PRF	A transcriptional repressor of c-myc	6.00
Ets	v-ets erythroblastosis virus E26 oncogene homolog 1	5.20
SIE	Serum inducible element	4.54
SMAD/SBE	MADH: MAD, mothers against decapentaplegic homolog	4.31
PEBP-2	Polyomavirus enhancer-binding protein 2	4.28
LXRE-1	Liver X receptor, nuclear receptor subfamily 1, group H, member 2	4.04
NFkB	Nuclear factor of kappa light polypeptide gene enhancer in B-cells 1	3.33
HOXA-4	Homeobox A4	3.00
EGR	Early growth response element	2.94
NF-1	Neurofibromatosis-related protein NF-1	2.90
PPUR	Purine-rich sequences binding sequence	2.89
SPERM-1	A Pou domain gene transiently expressed immediately prior to meiosis I in the male germ cell	2.82
NF-1/L	A member of the CTF/NF-1 transcription factor family	2.78
Ets/PEA3	ETS-domain transcription factor pea3	2.25
MTF	MRE-binding transcription factor-1	2.23
MBP-1	HIV-EP1; MHC-binding protein 1	2.17
RFX-1/2/3	A transactivator of hepatitis B virus enhancer I	2.14
LF-B2	Liver-specific factors	2.06

**Table 2:** Transcription factors with decreased binding activities in response to Cd treatment. HK-2 cells were treated with 40 µM Cd in serum-free culture medium for 3 h. The cells were separated into nuclear and post-nuclear fractions. The protein/DNA binding array was performed using the Combo Protein/DNA Array. Spot density was evaluated using ImageQuantTL software. Transcription factors whose activities were decreased 0.5-fold or less are listed.

Name	Description	Ratio
CEA	Carcinoembryonic antigen gene promoter	0.09
HIF-1	Hypoxia-inducible factor 1	0.12
GATA-1	GATA binding protein-1	0.19
EGR	Early growth response element	0.21
MT-Box	Tentative new binding domain, located in hTERT promotoer	0.21
MZF1	Myeloid zinc finger 1	0.22
POU2F3	Octamer-binding site in epidermis (POU domain factor)	0.23
PPAR	Peroxisome proliferator activated receptor alpha	0.27
CEF-2	cTnC (Slow/Cardiac Troponin C)	0.27
MEF2A	MADS box transcription enhancer factor 2	0.29
TEF1	Transcription enhancer factor-1	0.29
MSP-1	SAA with SP1 binding site removed	0.31
Sp-1	Sp1 transcription factor	0.32
ARNT	Aryl hydrocarbon receptor nuclear translocator binding element	0.34
Myb	Myb proto-oncogene protein	0.34
GATA-3	GATA binding protein-3	0.36
HOXD-9	Homeobox D9	0.36
GATA-2	GATA binding protein-2	0.37
L-III BP	Pyruvate kinase L gene binding protein III	0.38
PAX-6	Paired box protein Pax-6	0.39
Antioxidant RE	Antioxidant responsive element	0.40
AP-3	Activator protein 3	0.40
GATA-6	GATA binding protein-6	0.42
PAX-8	Paired box protein Pax-8	0.42
SAA	Amyloid precursor protein (APP) regulatory element	0.42
CTCF	CCCTC binding factor	0.42
PAX-4	Paired box protein Pax-4	0.46
FOXF1	Forkhead box F1a (HNF-3/Fkh Homolog-8)	0.50

### Identification of ARNT as a target transcription factor in Cd toxicity

**Figure 2:**
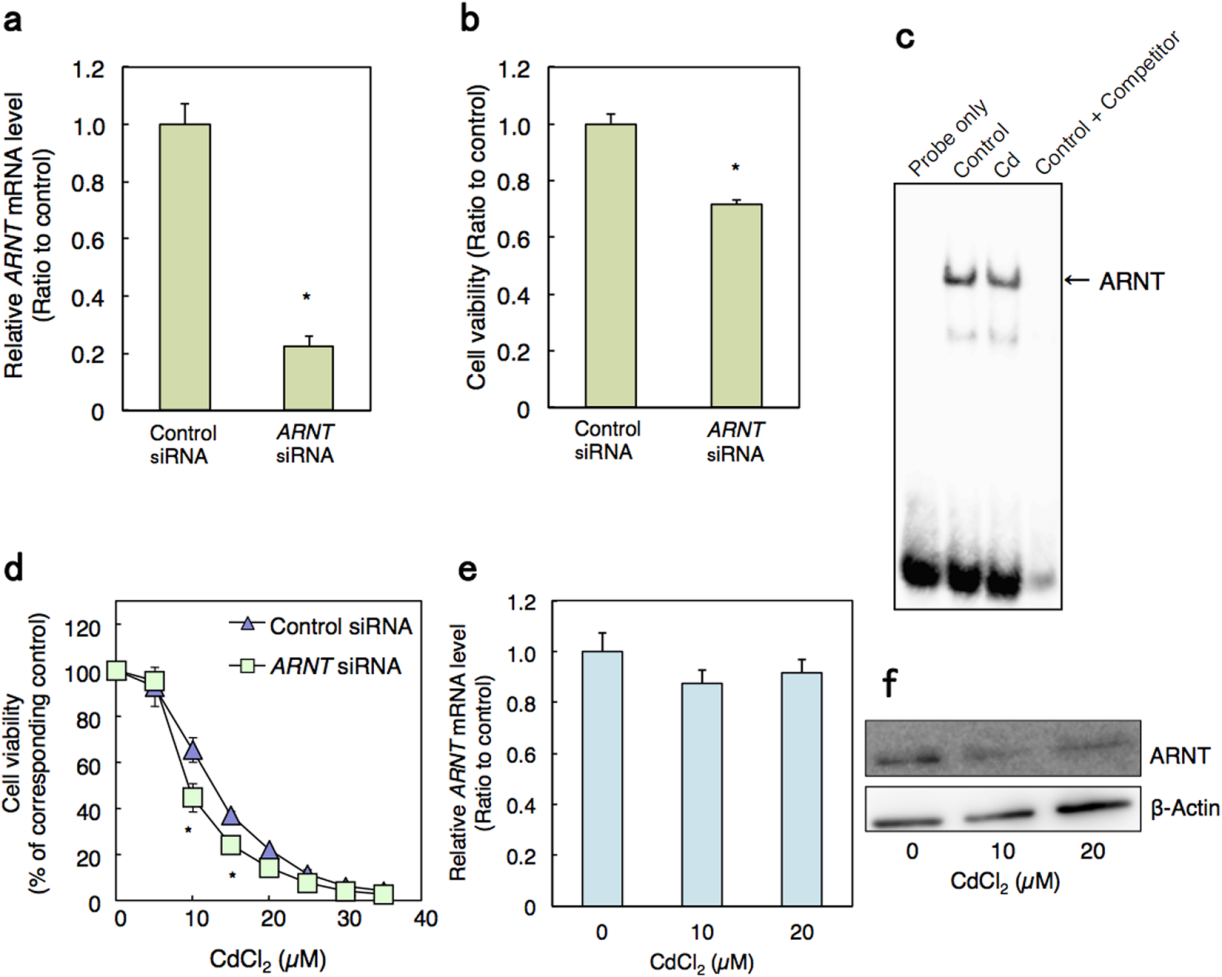
Association between Cd toxicity and DNA binding activity of ARNT transcription factor. (**a**) Knockdown efficiency of *ARNT* by siRNA treatment in HK-2 cells. HK-2 cells were transfected with control or *ARNT* siRNA for 24 h and *ARNT* mRNA levels were examined using real-time RT-PCR. mRNA levels were normalized with *GAPDH*. Values are the means ± S.D. (n = 3). (**b**) Cell viability of *ARNT* knockdown HK-2 cells. HK-2 cells were treated with control siRNA or *ARNT* siRNA for 24 h and MTT assays were performed. Values are the means ± S.D. (n = 4). *P < 0.05 vs. control. (**c**) DNA binding activity of ARNT in HK-2 cells treated with Cd. Nuclear extracts from control cells and HK-2 cells treated with 20 µM Cd for 6 h were used for the gel shift assay. (**d**) Viability of HK-2 cells with *ARNT* knockdown and treated with Cd. HK-2 cells were treated with control or 0.2 nM *ARNT* siRNA for 48 h, followed by treatment with Cd at the indicated concentrations for 24 h. Cell viability was examined using MTT assays. Values are the means ± S.D. (n = 4 or 5). *P < 0.05 vs. the corresponding control group. The absence of an error bar indicates that the S.D. was within the area of the symbol. (**e**) *ARNT* mRNA levels in Cd-treated HK-2 cells. HK-2 cells were treated with Cd at the indicated concentrations for 3 h. mRNA levels were examined using real-time RT-PCR and normalized with *GAPDH*. Values are the means ± S.D. (n = 3). (**f**) Cellular ARNT protein levels in HK-2 cells treated with Cd. Whole cell lysates of HK-2 cells treated with Cd for 6 h at the indicated concentrations were examined by western blot analysis with ARNT antibody. β-Actin was probed as a loading control. The blots were run under the same experimental conditions and cropped from same membrane. Uncropped images are provided in Supplementary Fig. 2b.

Our previous studies demonstrated that the suppression of gene expression is associated with Cd-induced renal toxicity^14,15,30,31^. These results imply that interruption in the regulation of gene expression is a key event in Cd toxicity. Therefore, we searched for a transcription factor that is involved in Cd toxicity, focusing on the transcription factors whose activities were decreased by Cd. In order to find the target transcription factor involved in Cd toxicity, it was determined whether the inhibition of expression of a transcription factor might affect the cell viability. As a result, each knockdown of six transcription factors triggered cell toxicity in HK-2 cells (Supplementary Table 1). Especially, the knockdown of *ARNT* [aryl hydrocarbon receptor nuclear translocator; known as hypoxia-inducible factor (HIF)-1β] by siRNA (Fig. 2a) conferred significant cell toxicity in HK-2 cells (Supplementary Table 1; Fig. 2b). EMSA assay showed that Cd treatment reduced the binding activity of ARNT (Fig. 2c). Knockdown of *ARNT* increased Cd toxicity in HK-2 cells (Fig. 2d), suggesting that decrease in the transcription activity of ARNT may strengthen Cd toxicity. In addition, although Cd did not affect the mRNA levels of *ARNT* (Fig. 2e), ARNT protein levels were decreased by Cd treatment in HK-2 cells (Fig. 2f). Together, this suggests that the ARNT transcription factor is a target in Cd-induced renal toxicity.

### BIRC3 as a responsible factor regulated by ARNT in Cd toxicity

**Figure 3:**
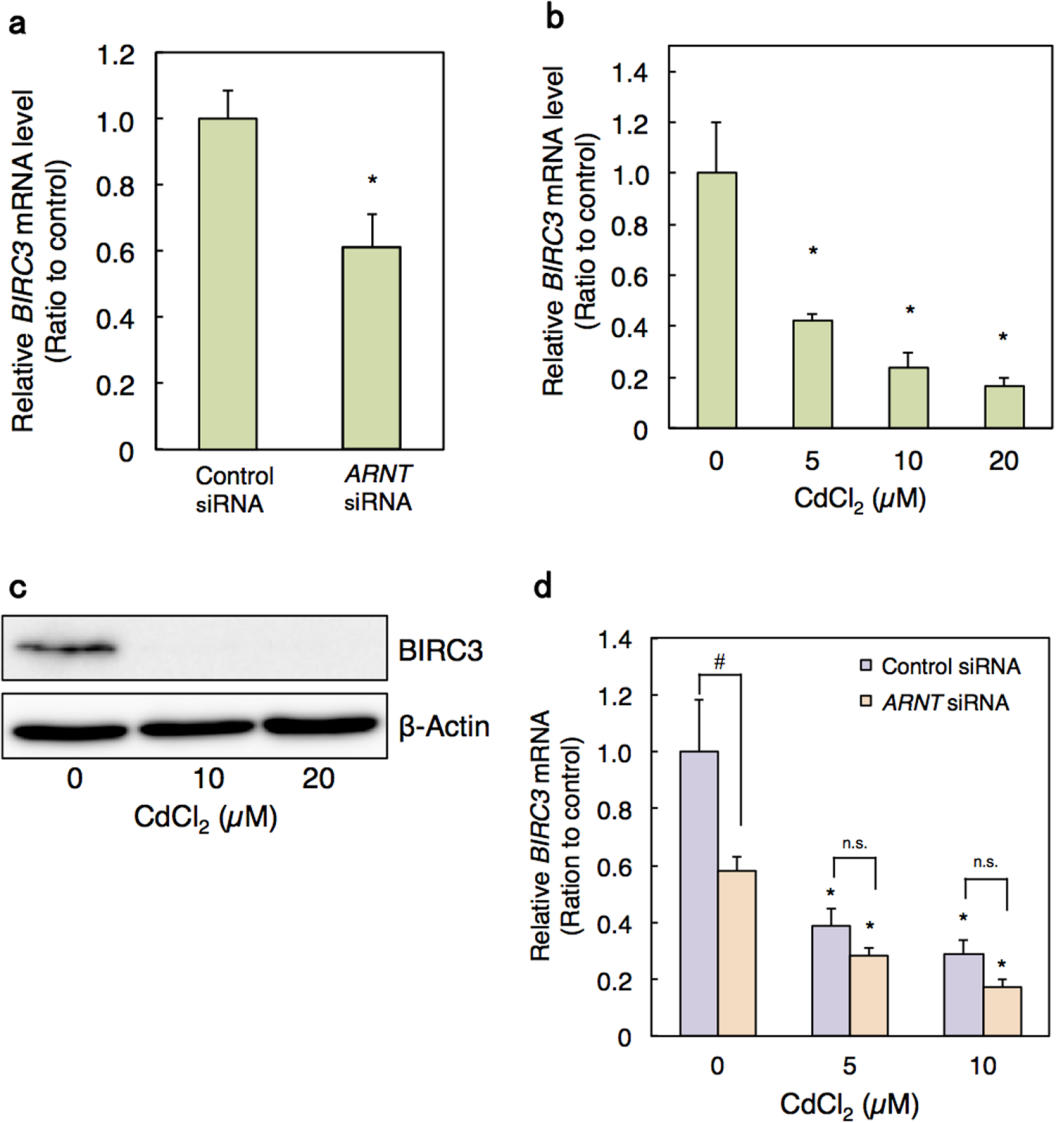
Decrease in intracellular BIRC3 level through the inhibition of ARNT activity in Cd-treated HK-2 cells. (**a**) *BIRC3* mRNA level in HK-2 cells treated with *ARNT* siRNA. HK-2 cells were transfected with control siRNA or *ARNT* siRNA for 48 h. mRNA levels were examined using real-time RT-PCR and normalized with *GAPDH*. Values are the means ± S.D. (n = 3). (**b**) *BIRC3* mRNA level in HK-2 cells treated with Cd. HK-2 cells were grown in 6-well plates for 48 h and treated with the indicated concentrations of Cd for 6 h. mRNA levels were examined using real-time RT-PCR and normalized with *GAPDH*. Values are the means ± S.D. (n = 3). (**c**) BIRC3 protein levels in HK-2 cells treated with Cd. Western blot analysis was performed for BIRC3 in HK-2 cells treated with the indicated concentrations of Cd for 6 h. β-Actin was probed as a loading control. The blots were run under the same experimental conditions and cropped from same membrane. Uncropped images are provided in Supplementary Fig. 2c. (**d**) *BIRC3* mRNA level in Cd-treated *ARNT* knockdown HK-2 cells. HK-2 cells were transfected with control or *ARNT* siRNA for 48 h and then treated with Cd for 6 h. mRNA levels were examined using real-time RT-PCR and normalized with *GAPDH*. Values are the means ± S.D. (n = 3). *P < 0.05 vs. corresponding control group; ^#^ *P* < 0.05 vs. control siRNA group.

We next examined which downstream factor of ARNT may be involved in Cd toxicity. Because ARNT binding activity was decreased by Cd and the knockdown of *ARNT* by siRNA decreased cell viability, we performed DNA microarray analysis to determine the gene expressions that were decreased in *ARNT* knockdown HK-2 cells. The results showed that the expressions of 27 genes were decreased by less than 0.5 fold in *ARNT* knockdown cells (Table 3). Interestingly, real-time RT-PCR showed that the gene expression of *BIRC3* [baculoviral IAP (inhibitor of apoptosis protein) repeat containing 3; known as cIAP2], an apoptosis inhibitor, was decreased by both *ARNT* siRNA as well as Cd treatment (Fig. 3a,b). Moreover, the protein level of BIRC3 was drastically decreased by Cd treatment in HK-cells (Fig. 3c). To investigate whether the Cd-induced decrease in *BIRC3* gene expression was mediated *via* ARNT, *BIRC3* mRNA levels were examined in *ARNT* knockdown HK-2 cells treated with Cd (Fig. 3d). *BIRC3* mRNA levels were decreased by Cd treatment in both control and *ARNT* knockdown cells. However, the significant decrease in *BIRC3* gene expression by *ARNT* knockdown without Cd was vanished upon Cd treatment. To determine the significance, we performed statistical analysis (Supplementary Table 2). Even with the *F*(0.95) of siRNA*Cd as 3.89, the *F* value of 6.92 is within the rejection region. Moreover, the *P* value of siRNA*Cd is below 0.05. Therefore, the siRNA effect and Cd treatment effect is significantly dependent. These results suggest that Cd treatment reduces cellular BIRC3 level through the suppression of ARNT transcription activity.

**Table 3:** Downregulated genes in HK-2 cells transfected with *ARNT* siRNA (<0.5-fold).

Accession Number	Gene Name	Description	Ratio
NM_152417	*TMEM68*	Transmembrane protein 68	0.219
NM_006243	*PPP2R5A*	Protein phosphatase 2, regulatory subunit B’, alpha	0.314
NM_031885	*BBS2*	Bardet-Biedl syndrome 2	0.373
NM_003003	*SEC*. *14L1*	SEC. 14-like 1 (S. cerevisiae)	0.376
NM_080605	*B3GALT6*	UDP-Gal:betaGal beta 1,3-galactosyltransferase polypeptide 6	0.393
NM_017901	*TPCN1*	Two pore segment channel 1	0.398
NM_020422	*TMEM159*	Transmembrane protein 159	0.421
NM_021158	*TRIB3*	Tribbles pseudokinase 3	0.421
NM_033308	*ABCA7*	ATP-binding cassette, sub-family A (ABC1), member 7	0.423
NM_004199	*P4HA2*	Prolyl 4-hydroxylase, alpha polypeptide II	0.432
NM_005178	*BCL3*	B-cell CLL/lymphoma 3	0.433
NM_145753	*PHLDB2*	Pleckstrin homology-like domain, family B, member 2	0.443
NM_006688	*C1QL1*	Complement component 1, q subcomponent-like 1	0.443
NM_006013	*RPL10*	Ribosomal protein L10	0.452
NM_016374	*ARID4B*	AT rich interactive domain 4B (RBP1-like)	0.454
NM_016096	*ZNF706*	Zinc finger protein 706	0.459
NM_018354	*TMEM74B*	Transmembrane protein 74B	0.467
NM_001165	*BIRC3*	Baculoviral IAP repeat containing 3	0.473
NM_014346	*TBC1D22A*	TBC1 domain family, member 22A	0.474
NM_006241	*PPP1R2*	Protein phosphatase 1, regulatory (inhibitor) subunit 2	0.475
NM_001541	*HSPB2*	Heat shock 27 kDa protein 2	0.480
NM_006896	*HOXA7*	Homeobox A7	0.482
NM_018155	*SLC25A36*	Solute carrier family 25 (pyrimidine nucleotide carrier), member 36	0.485
NM_001707	*BCL7B*	B-cell CLL/lymphoma 7B	0.487
NM_015062	*PPRC1*	Peroxisome proliferator-activated receptor gamma, coactivator-related 1	0.488
NM_016076	*DESI2*	Desumoylating isopeptidase 2	0.489
NM_018132	*CENPQ*	Centromere protein Q	0.492

**Figure 4:**
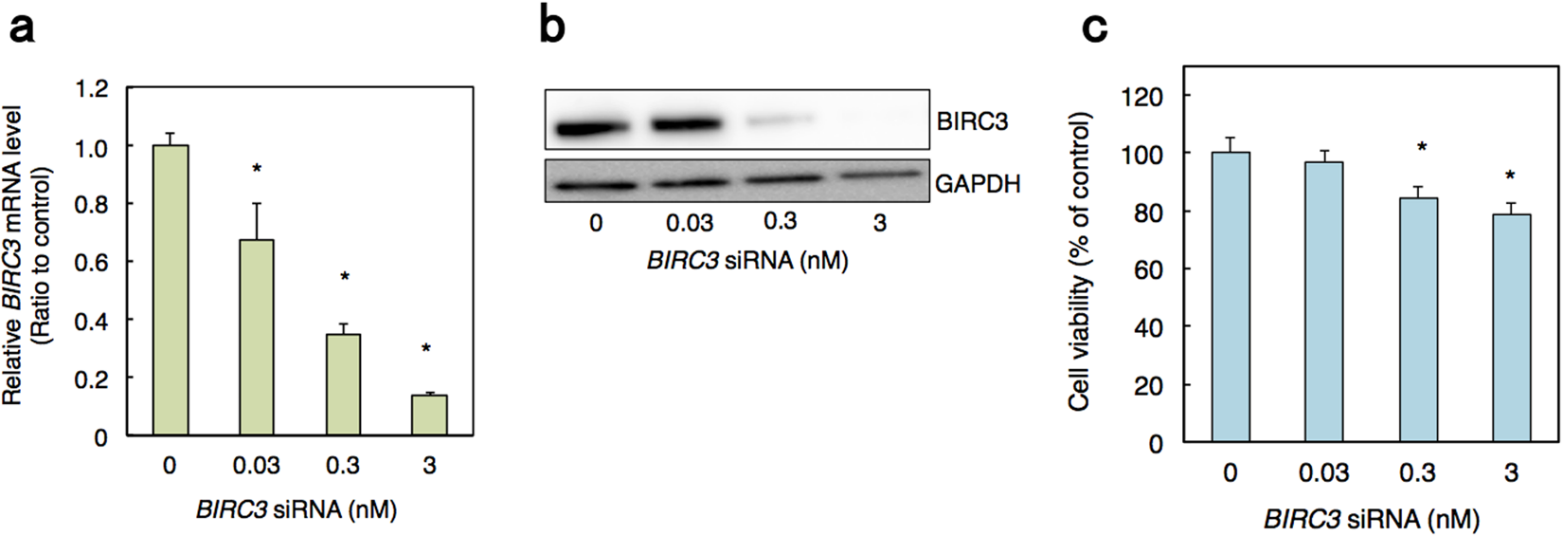
Decrease in cell viability by knockdown of *BIRC3*. (**a**) Knockdown efficiency of *BIRC3* by siRNA treatment in HK-2 cells. HK-2 cells were treated with control or *BIRC3* siRNA at the indicated concentration for 48 h. mRNA levels of *BIRC3* were examined using real-time RT-PCR. mRNA levels were normalized with *GAPDH*. Values are the means ± S.D. (n = 3). (**b**) BIRC3 protein levels in HK-2 cells treated with *BIRC3* siRNA. HK-2 cells were transfected with control or *BIRC3* siRNA at the indicated concentration for 48 h and then examined by western blot analysis for BIRC3 expression. GAPDH was probed as a loading control. The blots were run under the same experimental conditions and cropped from same membrane. Uncropped images are provided in Supplementary Fig. 2d. (**c**) Viability of HK-2 cells treated with *BIRC3* siRNA. HK-2 cells were treated with control or *BIRC3* siRNA at the indicated concentration for 48 h and examined using MTT assays. Values are the means ± S.D. (n = 3). *P < 0.05.

**Figure 5:**
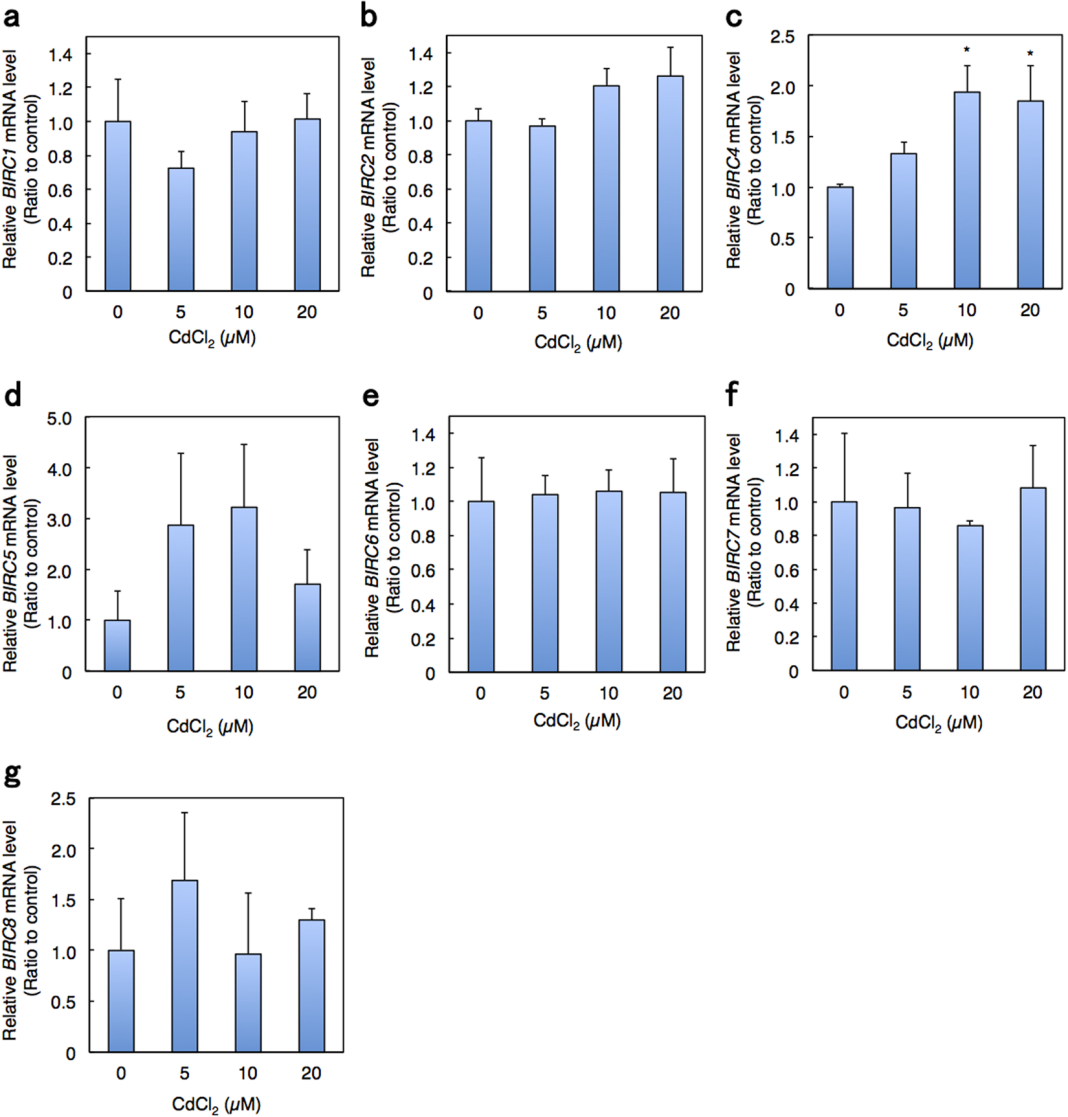
Effects of Cd on mRNA levels of BIRC family genes in HK-2 cells. HK-2 cells were grown in 6-well plates at a density of 250 cells/mm^2^ and cultured for 48 h. Culture medium was discarded and the cells were treated with Cd in serum-free culture medium for 6 h. mRNA levels of BIRC family genes were examined using real-time RT-PCR: (**a**) *BIRC1*, (**b**) *BIRC2*, (**c**) *BIRC4*, (**d**) *BIRC5*, (**e**) *BIRC6*, (**f**) *BIRC7*, and (**g**) *BIRC8*. mRNA levels were normalized with *GAPDH*. Values are the means ± S.D. (n = 3). *P < 0.05 vs. control group.

We next examined the impact of *BIRC3* knockdown on cell viability using siRNA transfection. The mRNA and protein levels of BIRC3 were decreased by siRNA in a dose-dependent manner (Fig. 4a,b). Moreover, *BIRC3* siRNA transfection significantly decreased HK-2 cell viability compared to control siRNA transfection cells (Fig. 4c). BIRC3 is a member of the BIRC family that is also known as the IAP family^32–34^. Eight family members have been identified in human, including BIRC1/NAIP, BIRC2/cIAP1, BIRC3/cIAP2, BIRC4/XIAP, BIRC5/Survivin, BIRC6/Apollon, BIRC7/ML-IAP and BIRC8/ILP^32–34^. To determine whether BIRC3 is specifically involved in Cd renal toxicity, we next examined the effect of Cd treatment on the mRNA levels of all BIRC family members (Fig. 5). Although the mRNA level of *BIRC4* was increased by Cd treatment, those of the other BIRC family members were unaffected by Cd treatment in HK-2 cells. These results suggest that specific reduction of BIRC3 levels by Cd-induced suppression of ARNT activity is involved in Cd renal toxicity.

### Cd-induced apoptosis through the suppression of BIRC3 expression

**Figure 6:**
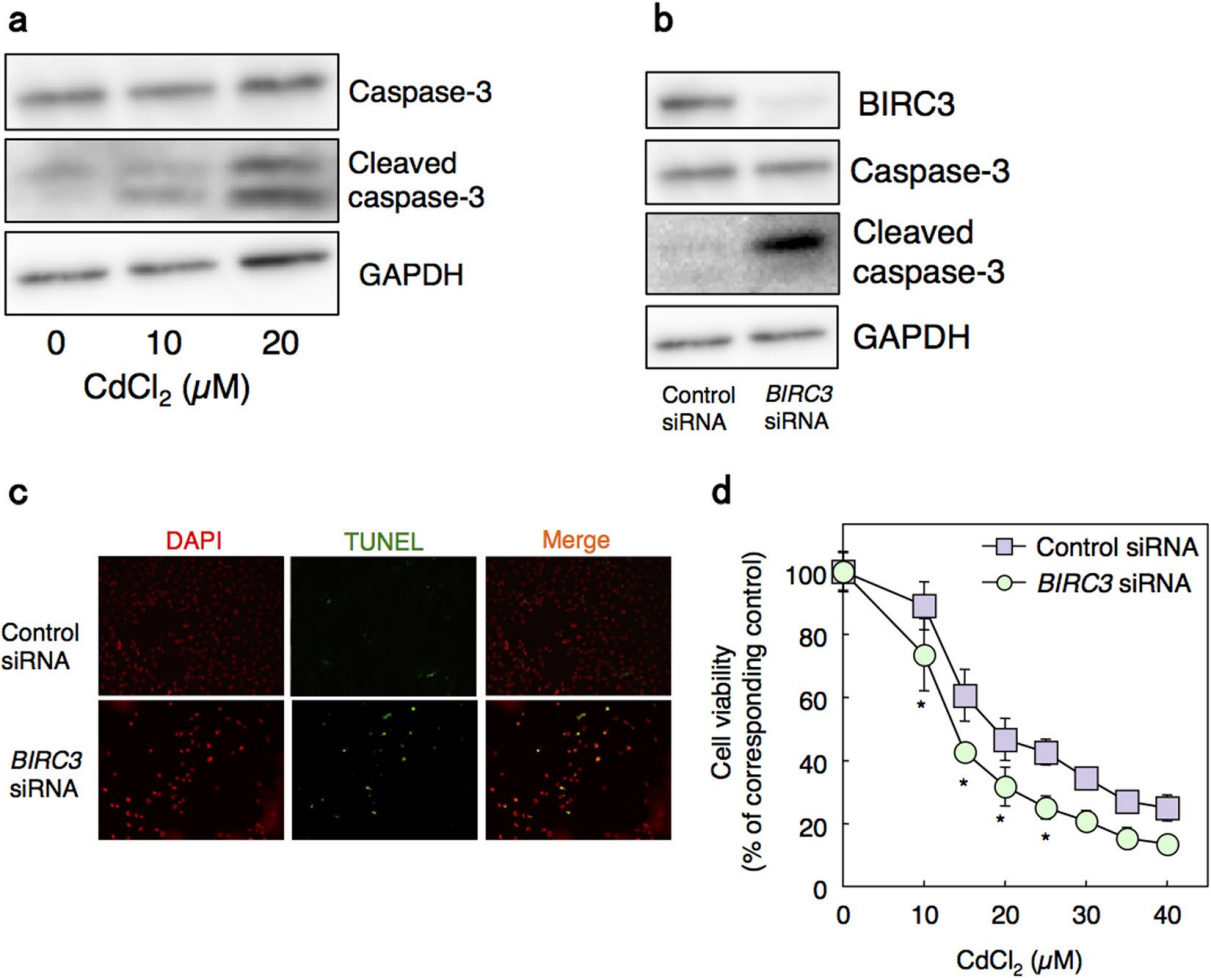
Apoptosis in *BIRC3* knockdown HK-2 cells. (**a**) Cleaved caspase-3 expression in HK-2 cells treated with Cd. HK-2 cells were grown in 60-mm dishes for 48 h and treated with Cd at the indicated concentration for 6 h. Western blot analysis was performed for caspase-3 and cleaved caspase-3. GAPDH was probed as a loading control. The blots were run under the same experimental conditions and cropped from same membrane. Uncropped images are provided in Supplementary Fig. 2e. (**b**) Cleaved caspase-3 expression in *BIRC3* knockdown HK-2 cells. Whole cell lysates of HK-2 cells treated with 0.3 nM *BIRC3* siRNA were used for western blot analysis for caspase-3 and cleaved caspase-3 expression. GAPDH was probed as a loading control. The blots were run under the same experimental conditions and cropped from same membrane. Uncropped images are provided in Supplementary Fig. 2 f. (**c**) Representative images of TUNEL assays in HK-2 cells with *BIRC3* knockdown. HK-2 cells were treated with control or 3 nM *BIRC3* siRNA for 48 h and TUNEL assays were performed. Nuclei were counterstained with DAPI, and the blue signal was converted to red (DAPI). Green fluorescein signals from TUNEL-positive cells (TUNEL) and red signals of nuclei were merged (Merge). Original magnification: × 100. (**d**) Viability of HK-2 cells with *BIRC3* knockdown and treated with Cd. HK-2 cells were treated with control or 0.3 nM *BIRC3* siRNA for 48 h, followed by treatment with Cd at the indicated concentrations for 12 h. Cell viability was examined using MTT assays. Values are the means ± S.D. (n = 4). *P < 0.05 vs. corresponding control group. The absence of an error bar indicates that the S.D. was within the area of the symbol.

Previous studies showed that Cd induces apoptosis in proximal tubular cells^8,14,15^, and that BIRC3 inhibits apoptosis through the activity of caspases^35^. Therefore, we next examined whether BIRC3 is involved in Cd-induced apoptosis. Consistent with published studies, Cd treatment increased the level of cleaved caspase-3, the active form of casapse-3, in HK-2 cells (Fig. 6a). Moreover, knockdown of *BIRC3* also increased the level of cleaved caspase-3 in HK-2 cells (Fig. 6b). These results suggest that knockdown of *BIRC3* may induce apoptosis. Therefore, we performed TUNEL assays and found that knockdown of *BIRC3* also induced apoptosis in HK-2 cells (Fig. 6c). Furthermore, knockdown of *BIRC3* significantly increased Cd toxicity in HK-2 cells (Fig. 6d). Together, this demonstrates that a decrease in BIRC3 is involved in Cd-induced apoptosis in HK-2 cells.

### Gene expression of BIRC3 and the activation of caspase-3 in several cultured cells

**Figure 7:**
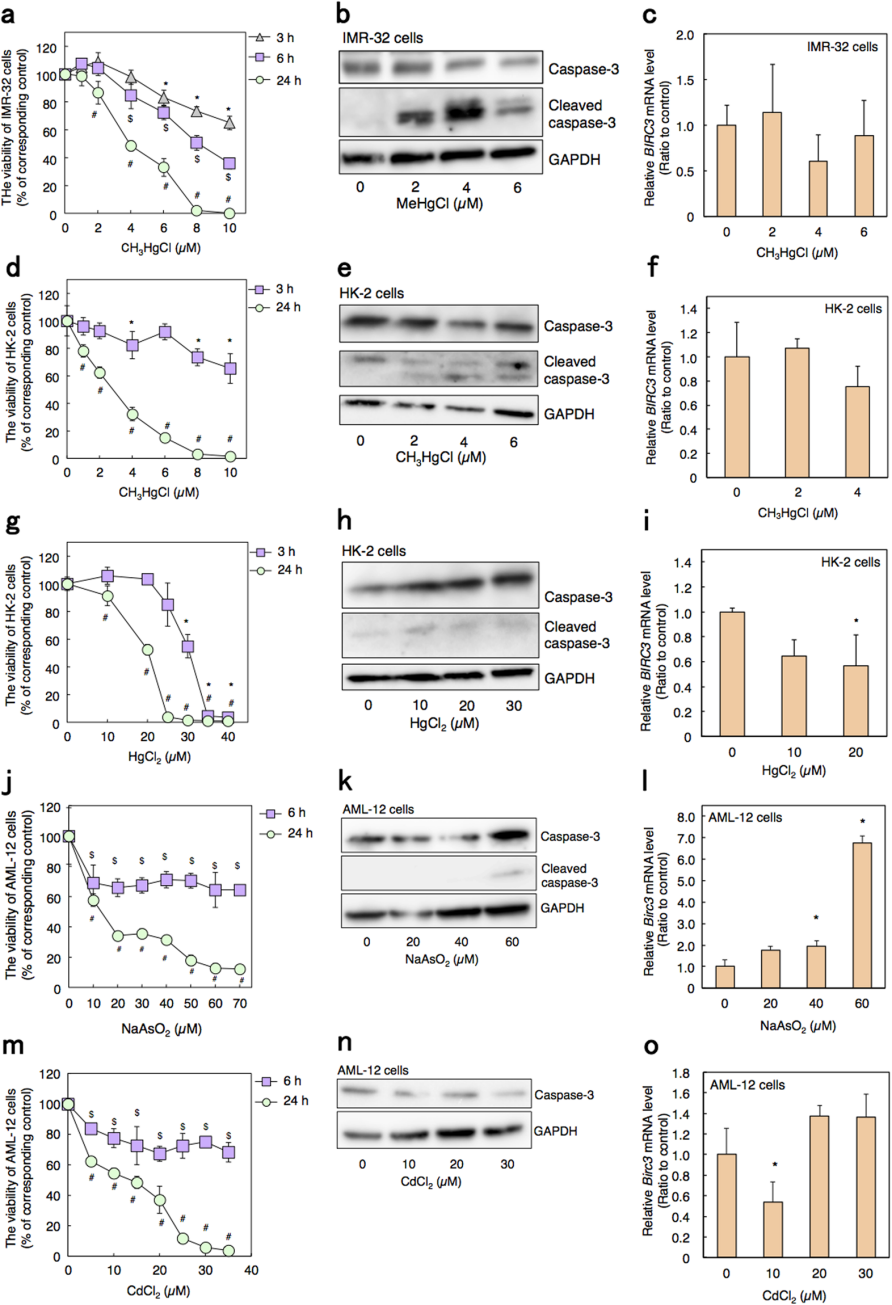
Effect of various toxic metal(loid)s on caspase activity and *BIRC3* mRNA levels. Cell viability of IMR-32 cells treated with methylmercury (CH_3_HgCl) for indicated time (**a**). Cleaved caspase-3 expression (**b**) and the mRNA level of *BIRC3* (**c**) of IMR-32 cells treated with methylmercury for 6 h. Cell viability of HK-cells treated with methylmercury for indicated time (**d**). Cleaved caspase-3 expression (**e**) and the mRNA level of *BIRC3* (**f**) of HK-cells treated with methylmercury for 3 h. Cell viability of HK-2 cells treated with inorganic mercury (HgCl_2_) for indicated time (**g**). Cleaved caspase-3 expression (**h**) and the mRNA level of *BIRC3* (**i**) of HK-2 cells treated with inorganic mercury for 3 h. Cell viability of AML-12 cells treated with arsenic (NaAsO_2_) for indicated time (**j**). Caspase-3 expression (**k**) and the mRNA level of *Birc3* (**l**) of AML-12 cells treated with arsenic for 6 h. Cell viability of AML-12 cells treated with Cd for indicated time (**m**). Caspase-3 expression (**n**) and the mRNA level of *Birc3* (**o**) of AML-12 cells treated with Cd for 6 h. (**a**, **d**, **g**, **j**, **m**) Cell viabilities were examined using MTT assays after the treatment with each toxic metal(loid) for the indicated times. Values are the means ± S.D. (n = 5). *Significantly different from the control group of 3 h treated group, *P* < 0.05. ^$^Significantly different from the control group of 6 h treated group, *P* < 0.05. ^#^Significantly different from the control group of 24 h treated group, *P* < 0.05. The absence of an error bar indicates that the S.D. was within the area of the symbol. (**b**, **e**, **h**, **k**, **n**) Whole cell lysates were used for western blot analysis and probed with caspase-3 or cleaved caspase-3 antibody. GAPDH was probed as a loading control. The blots were run under the same experimental conditions and cropped from same membrane. Uncropped images are provided in Supplementary Fig. 3. (**c**, **f**, **i**, **l**, **o**) mRNA level of *BIRC3* or *Birc3* was examined using real-time RT-PCR. mRNA levels were normalized with *GAPDH* or *β-actin*. Values are the means ± S.D. (n = 3). *P < 0.05 vs. control.

In addition to Cd, other various metal(loid) toxicants also induce apoptosis in cultured cells. Methylmercury and inorganic mercury induce apoptosis in neuroblastoma and kidney cells, respectively^36–38^. The arsenic compound also causes apoptosis in hepatic cells^39,40^. To determine the specificity of BIRC3 function, we next examined the changes in *BIRC3* gene expression and cleaved caspase-3 levels in several cell lines treated with metal(loid)s. Under the condition that methylmercury decreased the viability of human neuroblastoma (IMR-32 cells) by 20%, methylmercury increased cleaved caspase-3 levels; on the other hand, mRNA level of *BIRC3* was not changed by methylmercury treatment (Fig. 7a–c). In HK-2 cells, methylmercury decreased cell viability by 10% as well as increased cleaved caspase-3 levels, but had no effect on *BIRC3* mRNA levels (Fig. 7d–f). Inorganic mercury decreased *BIRC3* mRNA levels in HK-2 cells even when the viability was almost the same as the control group; on the other hand, although inorganic mercury treatment reduced viability of HK-2 cells to 60% compared to control, cleaved caspase-3 levels were unchanged (Fig. 7g–i). In mouse hepatic cells (AML-12 cells), arsenic decreased the cell viability by 30%, with a significant increase in the mRNA level of *Birc3* (Fig. 7j,l). Western blot analysis showed the protein levels of caspase-3 and cleaved caspase-3 were slightly increased by arsenic treatment (Fig. 7k). Although 6 h of Cd treatment (10 to 30 µM) decreased AML-12 cell viability to approximately 30%, only 10 µM Cd treatment decreased *Birc3* mRNA levels (Fig. 7m,o). No cleaved caspase-3 levels were detected in AML-12 cells treated with Cd (Fig. 7n). As the western blot analysis did not clearly show the band of cleaved caspase-3 protein, we confirmed the analysis using staurosporin, an apoptosis inducer. Staurosporin drastically increased the protein level of cleaved caspase-3 in AML-12 cells (Supplementary Fig. 1). These data suggest that the activation of caspase-3 by suppression of *BIRC3* gene expression by Cd treatment may mainly occur in proximal tubular cells.

## Discussion

**Figure 8:**
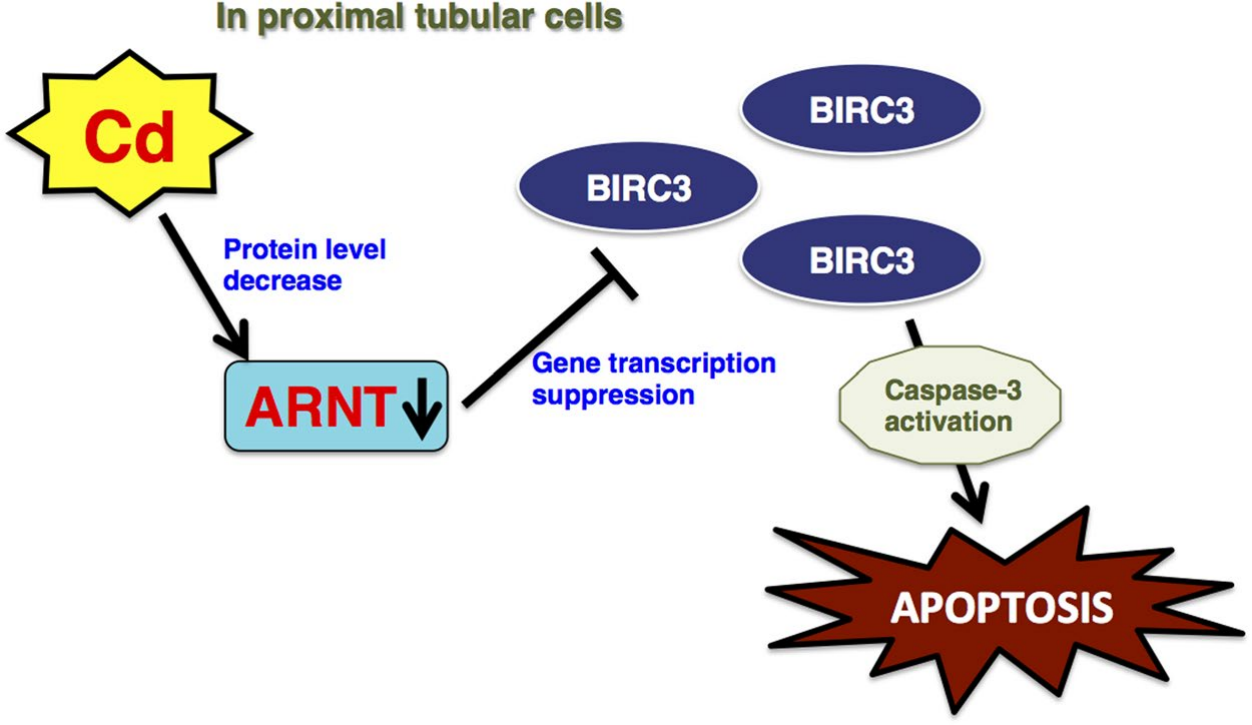
Scheme of Cd toxicity through the ARNT-regulated BIRC3 related apoptosis in proximal tubular cells. Cd suppresses the activity of ARNT transcription factors through the decrease of cellular protein level. The suppressed transcription activity decreases the expression of *BIRC3*. Down-regulation of *BIRC3* expression by inhibition of ARNT activates caspase-3.

Here we have found that Cd induces apoptosis through the quantitative alleviation of apoptosis inhibitor, BIRC3, through the suppression of its transcription in human proximal tubular cells. Recent studies have reported that chronic Cd exposure can induce apoptosis in renal cells^8,41–46^, and Cd induces *via* an ER-mediated pathway and mitochondrial-mediated pathway. In porcine renal proximal tubular epithelial LLC-PK1 cells, Cd induces ER stress and causes the activation of the unfolded protein response (UPR)-dependent apoptotic pathway, such as the IRE1-XBP1-JNK pathway^45,46^. In rat proximal tubule WKPT-0293 Cl.2 cells and mouse renal mesangial cells, Cd stimulates the release of pro-apoptotic factors from mitochondria^41,44^. Cd induces the swelling of mitochondria and subsequent cytochrome *c* release^42,43^. Our recent studies demonstrated that Cd induces apoptosis through p53 overaccumulation in human and rat proximal tubular cells^13–15^. In p53-mediated apoptosis, Cd inhibits the degradation of p53 by suppression of the gene expression of the UBE2D family, which is an E2 family enzyme in the UPS^13–15^. In the present study, we suggest a new apoptotic pathway involved in Cd toxicity. To the best of our knowledge, the ARNT-BIRC3 pathway is the first elucidated mechanism involved in Cd-induced apoptosis in proximal tubular cells (Fig. 8).

Our findings demonstrate that Cd suppresses the binding activity of the ARNT transcription factor in regulating *BIRC3* expression. Our previous reports revealed that transcription factors FOXF1 and YY1 are involved in the pathway by which Cd decreases the expression of genes coding for UBE2D proteins^14,27^. Therefore, together these results suggest that Cd induces apoptosis through the inhibition of anti-apoptosis proteins as well as the promotion of pro-apoptosis proteins. This also suggests that the initiation pathway responsible for Cd-induced apoptosis is the inhibition of transcription activity. Many studies have examined Cd toxicity, however only a few studies have reported the transcript pathway involved in Cd toxicity. A recent study reported that phosphorylation of the transcription factor FOXO3 promotes cell survival upon Cd treatment^47^. The upregulation of the transcription factor Snail by the activation of Notch1 signaling is reported to be involved in the Cd-induced decrease in cell-cell adhesion^48^. In this study, we propose a novel transcription factor ARNT in driving HK-2 cells to apoptotic cell death.

Our protein/DNA binding array analysis in this study revealed that the binding activities of 48 transcription factors were affected by Cd treatment. Our previous study determined that Cd decreased the activity of the FOXF1 transcription factor, which suppressed the downstream factor *UBE2D4*, leading to apoptosis through p53 overaccumulation^14^. Our results identified FOXF1 as one of the transcription factors whose activities were decreased by Cd treatment (Table 2). In Supplementary table 1, the list of transcription factors whose gene knockdown affects cell viability is shown. In addition to ARNT and FOXF1, such transcription factors as GATAs and MEF2A may play essential roles in the pathway of Cd toxicity. Moreover, an increase in DNA binding activity of the apoptotic-related transcription factor p53, which was accumulated in proximal tubular cells by Cd, was detected in our protein/DNA binding array upon Cd treatment (Table 1). Thus, the results from our protein/DNA binding array can propose useful and valid information to help elucidate transcription factors involved in Cd toxicity.

ARNT is a member of the basic helix-loop-helix/Per-ARNT-Sim family. Previous studies showed that ARNT heterodimerizes with the aryl hydrocarbon receptor (AhR) or oxygen sensitive alpha subunit (HIF-1α or HIF-2α)^49–51^. AhR is a ligand-binding protein that functions as the response factor to environmental pollutant exposure, including ligands such as benzo[*a*]pyrene and 2,3,7,8-tetrachlorodibenzo-*p*-dioxin^52,53^. The non-ligand bound AhR binds instead to two heat shock proteins (XAP2 and HSP90) and the cochaperone p23 in the cytosol^52^. Ligand binding disrupts the complex and causes AhR to translocate into the nucleus. After heterodimerization with ARNT, AhR/ARNT binds to xenobiotic response elements (XREs) or dioxin responsive elements (DREs) in the promoter sequences of target genes^52^. In normoxia, HIF-1α and HIF-2α are hydroxylated and rapidly degraded by the UPS^50,54^. In hypoxia, HIF-1α and HIF-2α are stabilized and bind to ARNT to regulate the expression of downstream target genes^50,54^. HIF-mediated pathways are essential cellular responses to hypoxia such as angiogenesis, erythropoiesis, cell growth, and cell differentiation^54–57^. Nonetheless, previous studies suggested that independent of its role in AhR and HIF signaling, ARNT has new regulatory functions in the expression of cyclooxygenase-2, 12(*S*)-lipoxygenase, and p21 genes under normoxia conditions^58–60^. In addition, ARNT is associated with the proliferation and survival of tumor cell lines by regulating cellular processes^49,58^. These observations, including our findings, indicate that ARNT activity is not only induced by such signals as AhR and HIF pathway but is also suppressed by signals apart from the AhR or HIF pathway. In this study, HIF-1 activity was decreased by Cd treatment (Table 2); therefore, Cd-mediated suppression of ARNT activity may involve the AhR and HIF pathway. Further studies are required to elucidate whether the AhR or HIF pathway may be associated with Cd-suppressed ARNT activity.

Apoptosis is a highly programmed pathway of cell death that is typically achieved through the activation of caspases^35^. During death receptor- or mitochondrial-mediated apoptosis, initiator caspases, such as caspase-8/9 and -10, are recruited *via* binding proteins FADD, cytochrome *c* and Apaf-1^61,62^. After recruitment of initiator caspases, effector caspases such as caspase-3 and -7 are activated by initiator caspases following cleavage into large and small subunits^63,64^. Cells have evolved important mechanisms to regulate caspase activity, for example, using the BIRC family. BIRC family members possess one or more baculorvirus IAP repeat (BIR) domains that selectively inhibits the activity of caspase-9, -3, or -7^32–34^. BIRC4, also known as XIAP (X-linked IAP), is the most characterized member of the family^35^. BIRC4 blocks apoptosis by inhibiting caspases, using the interaction its BIR2 and BIR3 regions with active-site pocket of caspases^65^. Based on the mechanism of BIRC4, most BIRC family members were suggested to neutralize caspase activities in the same manner. However, recent studies have reported that BIRC family members are functionally non-equivalent and regulate caspase activities *via* distinct mechanisms^66–68^. BIRC2, BIRC3 and BIRC4 contain a C-terminal RING zinc finger domain with E3 ubiquitin ligase activity that mediates proteasomal degradation of cellular targets as well as themselves^69^. BIRC2 was demonstrated to interact with caspase-7 independently on the active site pocket^66^. BIRC2 ubiquitinates active caspase-3 and -7 and mediates their proteasomal degradation, thereby suppressing apoptosis^67^. In this study, the decrease in BIRC3 protein level increased the level of the active form of caspase-3. This suggests that BIRC3 may be directly involved in degradation of the active form of caspase-3 rather than interrupting the active site of caspase-3 in HK-2 cells. Further studies are required for elucidating the precise mechanism underlying BIRC3-mediated caspase-3 deactivation upon Cd treatment.

Our most striking observation in this study is our novel finding, to the best of our knowledge, that the ARNT-BIRC3 pathway is involved in Cd-induced apoptosis in proximal tubular cells. The regulation of *BIRC3* gene expression has been previously unknown; however, our study suggests that ARNT may be a key transcription factor in BIRC3 regulation. Finally, we provide valuable information about the critical transcription factors for elucidating the toxic and defense mechanism pathways in response to Cd.

## Methods

### Cell culture and treatment

Human proximal tubular cells (HK-2 cells) were purchased from ATCC (Manassas, MA, USA), and cultured in Dulbecco’s Modified Eagle’s Medium/Nutrient Mixture F-12 Ham (DMEM/F-12) (Sigma-Aldrich, St. Louis, MO, USA), supplemented with 10% fetal bovine serum (FBS) (Gibco, Grand Island, NY, USA), 25 U/mL penicillin (DS Pharm, Osaka, Japan), 25 µg/mL streptomycin (DS Pharm), 1% Insulin-Transferrin-Selenium-X (Gibco), 10 ng/mL EGF (epidermal growth factor; Sigma-Aldrich), and 5 ng/mL hydrocortisone at 37 °C in a humidified incubator containing 5% CO_2_.

Human neuroblastoma cells (IMR-32 cells) were kindly provided from Dr. Katsuhiko Muraki (Laboratory of Cellular Pharmacology, School of Pharmacy, Aichi Gakuin University, Japan). IMR-32 cells were cultured in DMEM supplemented with 10% FBS, 25 U/mL penicillin, 25 µg/mL streptomycin, and 1% MEM Non-essential Amino Acid Solution (Sigma-Aldrich) at 37 °C in a humidified incubator containing 5% CO_2_.

Mouse normal hepatocytes (AML12 cells) were purchased ATCC, and cultured in DMEM/F-12, supplemented with 10% FBS, 1 mM sodium pyruvate (Gibco), 25 U/mL penicillin, 25 µg/mL streptomycin, 1% Insulin-Transferrin-Selenium-X, and 10 ng/mL EGF at 37 °C in a humidified incubator containing 5% CO_2_.

HK-2 cells and AML-12 cells were grown in plates at a density of 250 cells/mm^2^ and cultured for 48 h. IMR-32 cells were grown in plates at a density of 500 cells/mm^2^ and cultured for 48 h. The culture medium was discarded and the cells were treated with Cd (CdCl_2_; Wako Pure Chemical Industries, Osaka, Japan), methylmercury (CH_3_HgCl; GL Sciences Inc., Tokyo, Japan), inorganic mercury (HgCl_2_; Wako Pure Chemical Industries), or arsenic (NaAsO_2_; Wako Pure Chemical Industries) in serum-free culture medium for various times.

### Cell viability

HK-2 and AML-12 cells were grown in 96-well plates and cultured for 48 h. After treatment, culture medium was replaced with fresh 10% FBS-DMEM/F-12 containing MTT [3-(4,5-Dimethyl-2-thiazolyl)-2,5-diphenyl-2*H*-tetrazolium bromide; DOJINDO Laboratories, Kumamoto, Japan] and incubated for another 4 h at 37 °C. After removing the medium, 100 μL dimethyl sulfoxide (Wako Pure Chemical Industries) was added to MTT formazan. Absorbance at 570 nm was measured by the DTX880 multimode detector (Beckman Coulter Inc., Brea, CA, USA).

IMR-32 cells were grown in 96-well plates and cultured for 48 h. After treatment, 10% (w/v) Alamar Blue (Invitrogen, Grand Island, NY, USA) was added and incubated for 4 h at 37 °C. Fluorescence was measured at excitation wavelength of 540 nm and an emission wavelength of 595 nm by the DTX880 multimode detector.

### Western blot analysis

After treatment, cells were washed twice with ice-cold phosphate-buffered saline (PBS) (Nissui Pharmaceutical, Tokyo, Japan) and harvested in RIPA buffer [25 mM Tris (pH 7.6), 150 mM NaCl, 1% NP-40, 1% sodium deoxycholate 0.1% SDS; Thermo Fisher Scientific, Waltham, MA, USA]. Protein concentrations were measured using the BCA protein assay kit (Thermo Fisher Scientific). Protein samples were separated on SDS-polyacrylamide gels and transferred to a polyvinylidene fluoride membrane. The membrane was probed with primary antibodies and subsequently probed with horseradish peroxidase-conjugating (HRP) secondary antibodies (1:10000; GE Healthcare, Little Chalfont, UK). Proteins were detected by enhanced chemiluminescence using ImmunoStar® Zeta (Wako Pure Chemical Industries). The chemiluminescence images were taken using the LAS-500 or LAS-4000 (GE Healthcare) device. Primary antibodies were purchased as follows: GAPDH (1:2000) from American Research Products (Waltham, MA, USA); Lamin A/C (1:1000), ARNT (1:1000), BIRC3 (1:1000), caspase-3 (1:1000) and cleaved caspase-3 (1:1000) from Cell Signaling Technology (Danvers, MA, USA); and β-actin (1:1000) from Sigma-Aldrich.

### Nuclear extraction

Nuclei were extracted with the Nuclear Extraction Kit (Panomics; Affymetrix, Santa Clara, CA, USA). After Cd treatment, HK-2 cells were washed twice with ice-cold PBS and lysed on ice for 10 min in extraction buffer A including a protease inhibitor, phosphatase inhibitor, and dithiothreitol (DTT). The cells were harvested from the assay plates by scraping and pipetting up and down several times to disrupt the cell clumps. Nuclei were collected by centrifugation at 14,000 × *g* for 3 min at 4 °C. The pellet was resuspended in extraction buffer B including a protease inhibitor, phosphatase inhibitor, and DTT and incubated at 4 °C for 1 h. The mixture was then centrifuged at 14,000 × *g* for 5 min at 4 °C, and the supernatant was collected. Protein concentration was measured using the BCA protein assay kit.

### Protein/DNA binding array

HK-2 cells were grown in 60-mm dishes at a density of 250 cells/mm^2^ and cultured for 48 h. Culture medium was discarded and the cells were treated with 40 µM Cd in serum-free culture medium for 3 h. HK-2 cells were separated into nuclear and post-nuclear fractions. The protein/DNA binding array was performed using the Combo Protein/DNA Array (Affymetrix)^27^. In brief, 20 μg of nuclear extracts were mixed with a biotin-labeled probe mix, and the mixture was incubated at 15 °C for 30 min. The protein-bound probes in the mixture were isolated from the non-bound probes using a spin column. The protein-bound probes were eluted with column elution buffer and denatured at 95 °C for 3 min. The eluted probes were then added to the hybridization buffer and hybridized to the array membrane spotted with 345 consensus sequences complementary to the probes at 42 °C overnight. The membrane was washed twice in 2 × saline sodium citrate (SSC)/0.5% sodium dodecyl sulfate (SDS) at 42 °C for 20 min and then twice in 0.1 × SSC/0.5% SDS at 42 °C for 20 min. The membrane was then blocked with 1 × blocking buffer. The biotin-labeled probes were detected with streptavidin-horseradish peroxidase diluted 1:10000. The image was acquired using an LAS-4000 device. Spot density was evaluated using ImageQuantTL software (GE Healthcare).

### Gel shift assay

The gel shift assay was performed using the EMSA kit purchased from Panomics (Affymetrix). HK-2 cells were grown in 60-mm dishes at a density of 250 cells/mm^2^ and cultured for 48 h. After treatment, HK-2 cells were separated into nuclear and post-nuclear fractions. Nuclear protein (3 µg) was incubated with 10 ng DNA probe (biotin-labeled binding sequence to transcription factor) and 1 µg poly d(I-C) with binding buffer for 30 min at 15 °C in a thermal cycler (Takara Bio, Shiga, Japan). For the competition assay, 1,320 ng cold DNA probe was added. The protein-bound probe was electrophoresed on a 5.0% (w/v) TBE (Tris borate EDTA)-polyacrylamide gel in 0.5 × TBE buffer at 4 °C and then transferred to a Biodyne® B nylon membrane (Pall Corporation, Port Washington, NY, USA) in 0.5 × TBE buffer. The membrane was fixed by UV crosslinking (CL-1000 Ultraviolet Crosslinker; UVP, Upland, CA, USA) with 120 mJ/cm^2^. The membrane was blocked and probed with Streptavidin-HRP. The chemiluminescence images were taken using a LAS-3000 device.

### siRNA transfection

Silencer Select Pre-designed siRNAs were purchased from Ambion (Grand Island, NY, USA) as follows: s1613 and s1615 (Silencer® Select Pre-designed siRNA) for human *ARNT*; and s1451, s1452 and s1453 (Silencer® Select Pre-designed siRNA) for human *BIRC3*. Control siRNA (Silencer® Select Negative Control #1 siRNA) was also purchased from Ambion. siRNA transfection was performed using Lipofectamine RNAiMAX (Invitrogen). After the siRNA mixture was incubated for 15 min with Lipofectamine RNAiMAX and Opti-MEM® I Reduced Serum Medium (Opti-MEM; Gibco), HK-2 cells were transfected with the siRNA mixture (1 nM siRNA/sequence, 0.2% Lipofectamine RNAiMAX, 10% Opti-MEM).

### RNA extraction

Cells were washed twice with ice-cold PBS and total RNA was extracted with the PureLink^TM^ RNA Mini Kit (Ambion, Grand Island, NY, USA) and QuickGene-810 (Fujifilm). RNA quantification and purity was measured using BioSpec-nano (Shimadzu, Kyoto, Japan).

### DNA microarray

DNA microarray analysis was performed using previously described methods^11^. In brief, purified total RNA (5 μg) was applied to an OpArray Human V4.0 slide with 35,035 genes (Operon Technologies, Alameda, CA, USA). The Low RNA Input Fluorescent Linear Amplification Kit (Agilent Technologies, Santa Clara, CA, USA) was used to synthesize complementary RNA (cRNA) using double-stranded cDNA as a template. A primer containing a poly dT and a T7 polymerase promoter was annealed to poly A^+^RNA. Reverse transcriptase was then added to the reaction mix and double-stranded cDNA was transcribed from the untreated or siRNA-treated cells in the presence of cyanine (Cy) 3-CTP or Cy5-CTP (PerkinElmer, Waltham, MA, USA), respectively. These two sets of fluorescence-labeled cRNA were mixed and hybridized to an Operon microarray slide for 16 h at 42 °C using a Lucidea SlidePro Hybridizer (Amersham Bioscience, Little Chalfont, UK). Fluorescent images were recorded with CRBIO (Hitachi Software Engineering, Tokyo, Japan). Digitized image data were processed with DNASIS Array software (Hitachi Software Engineering). Following global normalization, the data were filtered to exclude genes with low-level expression. The intensity of the Cy5 (siRNA-treated) to Cy3 (control) ratio was calculated. Information on each gene was obtained from the National Center for Biotechnology Information (NCBI) database.

### Real time RT-PCR

Total RNA was incubated with the PrimeScript reverse transcription (RT) Reagent Kit (Perfect Real Time) (Takara Bio) to generate cDNA. Real-time PCR was performed with SYBR Premix Ex TaqII (Perfect Real Time) (Takara Bio) and the Thermal Cycler Dice Real Time System (Takara Bio). PCR conditions were as follows: 10 s hot-start at 95 °C followed by 40 cycles of 5 s at 95 °C and 30 s at 60 °C. Gene expression was normalized to *GAPDH* or *ß-actin* mRNA levels. The oligonucleotide sequences of the primers were as follows: sense, 5′-CTAGTGGCCATTGGCAGATT-3′, and antisense, 5′-CAATGTTGTGTCGGGAGATG-3′, for the human *ARNT* gene; sense,5′-CATGTGTGTGGAGGGTGAAG-3′, and antisense, 5′-TTTAACAGGGGACAGCATCC-3′, for the human *BIRC1* gene; sense, 5′-GCATTTTCCCAACTGTCCAT-3′, and antisense, 5′-ATTCGAGCTGCATGTGTCTG-3′, for the human *BIRC2* gene; sense, 5′-CAACAGATCTGGCAAAAGCA-3′, and antisense, 5′-TTGCTCAATTTTCCACCACA-3′, for the human *BIRC3* gene; sense, 5′-TGGGGTTCAGTTTCAAGGAC-3′, and antisense, 5′-TGCAACCAGAACCTCAAGTG-3′, for the human *BIRC4* gene; sense, 5′-GTTGCGCTTTCCTTTCTGTC-3′, and antisense, 5′-TCTCCGCAGTTTCCTCAAAT-3′, for the human *BIRC5* gene; sense, 5′-TGACGCTTTCAACCTCACTG-3′, and antisense, 5′-GTGTCCGCTGGACCAGTTAT-3′, for the human *BIRC6* gene; sense, 5′-TGGCCTCCTTCTATGACTGG-3′, and antisense, 5′-ACCTCACCTTGTCCTGATGG-3′, for the human *BIRC7* gene; sense, 5′-AAGCCCGGCTCATTACTTTT-3′, and antisense, 5′-ATCTTCCTTGGGCTTCCAGT-3′, for the human *BIRC8* gene; sense, 5′- GCACCGTCAAGGCTGAGAAC-3′, and antisense, 5′-TGGTGAAGACGCCAGTGGA-3′, for the human *GAPDH* gene; sense, 5′-TGACGTGTGTGACACCAATG-3′, and antisense, 5′-TGCTGCAGTGTTTCCTTTTG-3′, for the mouse *Birc3* gene; sense, 5′-CCTAAGGCCAACCGTGAAAA-3′, and antisense, 5′-AGCCATACAGGGACAGCACA-3′, for the mouse *ß-actin* gene.

### Detection of apoptosis

HK-2 cells were transferred to a Millicell EZ SLIDE (Millipore, Billerica, MA, USA) at a density of 250 cells/mm^2^ with the siRNA mixture (1 nM siRNA/sequence, 0.2% Lipofectamine RNAiMAX, 10% Opti-MEM) and incubated for 48 h. Apoptotic cells were detected using the *In Situ* Cell Death Detection Kit, Fluorescein (Roche, Mannheim, Germany) according to the manufacturer’s protocol. Fluorescence images were taken using a BIOREVO BZ-9000 microscope (Keyence, Osaka, Japan).

### Statistical analysis

Statistical analyses were performed using one or two-way analysis of variance (ANOVA). When the F value was significant (*P* < 0.05), Bonferroni’s multiple *t*-test was performed for post-hoc comparison (*P* < 0.05).

## Electronic supplementary material

Supplementary information
