# Supplementary material for: Identification of ARNT-regulated BIRC3 as the target factor in cadmium renal toxicity
Source: Sci Rep. 2017 Dec 11;7:17287. doi: 10.1038/s41598-017-17494-9 (PMC5725491; doi:10.1038/s41598-017-17494-9)

Supplementary Information of:

Identification of ARNT-regulated BIRC3 as the target factor in cadmium renal toxicity

Jin-Yong Lee[1], Maki Tokumoto[1], Gi-Wook Hwang[2], Moo-Yeol Lee[3] and Masahiko Satoh[1,*]

[1] Laboratory of Pharmaceutical Health Sciences, School of Pharmacy, Aichi Gakuin University, 1-100 Kusumoto-cho, Chikusa-ku, Nagoya, Aichi 464-8650, Japan

[2] Laboratory of Molecular and Biochemical Toxicology, Graduate School of Pharmaceutical Sciences, Tohoku University, Sendai 980-8578, Japan

[3] College of Pharmacy, Dongguk University, Goyang, Gyeonggi-do 410-820, Republic of Korea

[*] To whom correspondence should be addressed: Prof. Masahiko Satoh, Laboratory of Pharmaceutical Health Sciences, School of Pharmacy, Aichi Gakuin University, 1-100 Kusumoto-cho, Chikusa-ku, Nagoya, Aichi 464-8650, Japan, Telephone: +81-52-757-6790; FAX:+81-52-757-6799; E-mail: masahiko@dpc.agu.ac.jp

Supplementary Table 1. Effects of gene expression inhibition of transcription by siRNA on viability of HK-2 cells. HK-2 cells were treated with control or each siRNA against listed transcription factors for 24 or 48 h. After siRNA treatment, cell viability was examined using MTT assay. ↓↓↓, decrease more than 20%; ↓↓, decrease form 10% to 20%; ↓, decrease less than 10%; ↑↑, increase more than 10%; ↑, increase less than 10%; ±, no significant difference, compared to control siRNA group. The means of 24 h and 48 h are the time of siRNA treatment.

Name	Description	Cell viability	
		24 h	48 h
ARNT	Aryl hydrocarbon receptor/aryl hydrocarbon receptor nuclear translocator binding element	↓↓↓	↓↓
HIF-1	Hypoxia-inducible factor 1	↓	↑
GATA-1	GATA binding protein	↓↓↓	±
GATA-3	GATA binding protein	↓↓	↓↓
GATA-6	GATA binding protein	↓↓↓	↓↓
MEF2A	MADS box transcription enhancer factor 2A	↓↓↓	↓↓↓
FOXF1	Forkhead box F1a (HNF-3/Fkh Homolog-8)	↓↓	↓
Sp-1	Sp1 transcription factor	↓	±
PAX-4	Paired box protein Pax-4	±	±
PAX-6	Paired box protein Pax-6	↑	↑↑
PAX-8	Paired box protein Pax-8	↑	±
MZF1	Myeloid zinc finger 1	↑	±
Skn	Octamer-binding site in epidermis (POU domain factor)	↑	±
Myb	Myb proto-oncogene protein	↑	±
HOXD-9/10	Homeobox D9/10	↑	±
TEF1	Transcription enhancer factor-1	↑	±
CTCF	CCCTC binding factor	↑	↑
EGR	Early growth response element	±	±
PPAR	Peroxisome proliferator activated receptor alpha	±	±

Supplementary Table 2. *BIRC3* mRNA level in HK-2 cells treated with *ARNT* siRNA and/or Cd

HK-2 cells were treated with control or ARNT siRNA for 48 h. After siRNA treatment, HK-2 cells were treated with Cd for 6 h. mRNA levels were normalized with *GAPDH*. Statistical analysis was performed using two-factor factorial analysis of variance (ANOVA). S.D., standard deviation; SS, sum of squares; DF, degrees of freedom; MS, mean square.

BIRC3 mRNA Level						
	Control siRNA			*ARNT* siRNA		
Cd (μM)	0	5	10	0	5	10
Mean (normalized to *GAPDH*)	1.347001133	0.519876265	0.386710456	0.779472339	0.381038866	0.234515445
S.D.	0.242284834	0.081607362	0.066654882	0.072093856	0.033551716	0.03334809
Analysis of Variance						
Source	SS	DF	MS	*F* Value	*P* Value	*F*(0.95)
Total	2.624266721	17	-	-	-	-
siRNA	0.36856367	1	0.368563670	28.630043080	0.0001734	4.747225347
Cd	1.922994781	2	0.961497391	74.689162133	0.0000002	3.885293835
siRNA*Cd	0.178228444	2	0.089114222	6.922396925	0.0100196	3.885293835
Error	0.154479825	12	0.012873319	-	-	-

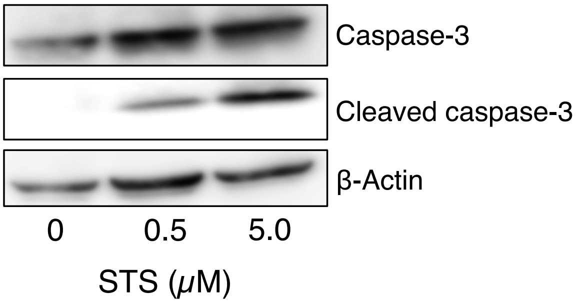

Supplementary Figure 1. Cellular protein level of cleaved caspase-3 in STS (staurosporine)-treated AML-12 cells. AML-12 cells were seeded in 6 cm plates at the density of $2.5 \times 10^4/cm^2$ and cultured for 48 h. The cells were treated with STS for 3 h. Whole cell lysates were used for western blot analysis and probed with cleaved caspase-3 or caspase-3 antibody. β-Actin was probed as a loading control. Uncropped images are provided in Supplementary Fig. 2c.

Supplementary Figure 2. Original images of cropped blots in figures 1-6.

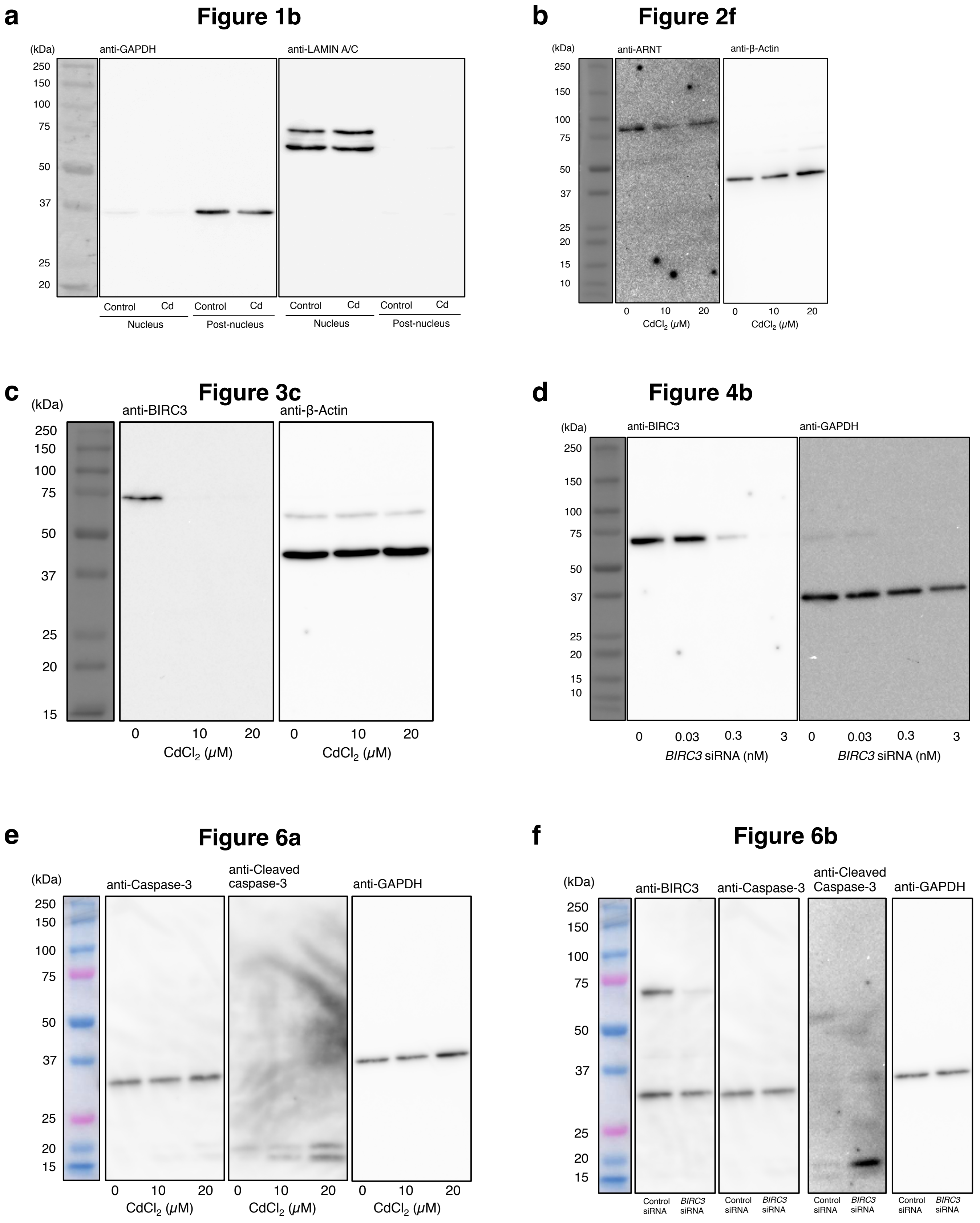

Supplementary Figure 3. Original images of cropped blots in figure 7.

a **Figure 7b**

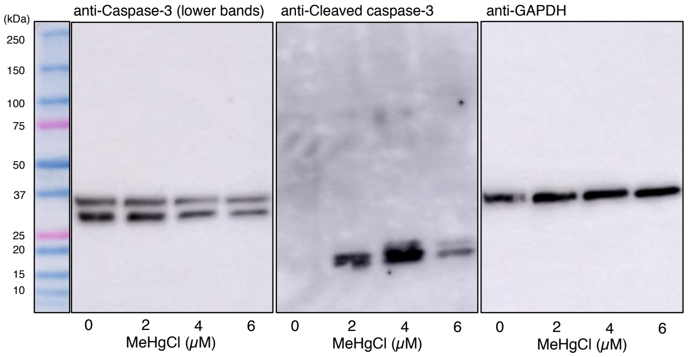

b **Figure 7e**

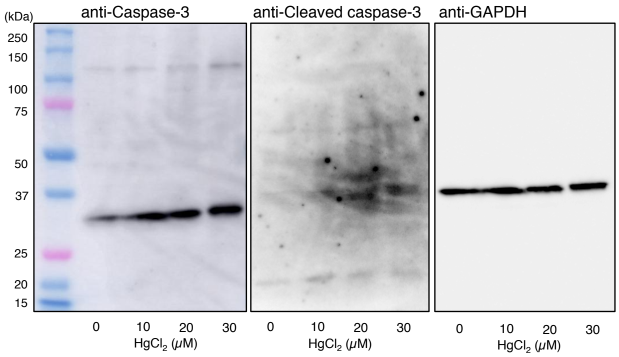

c **Figure 7h**

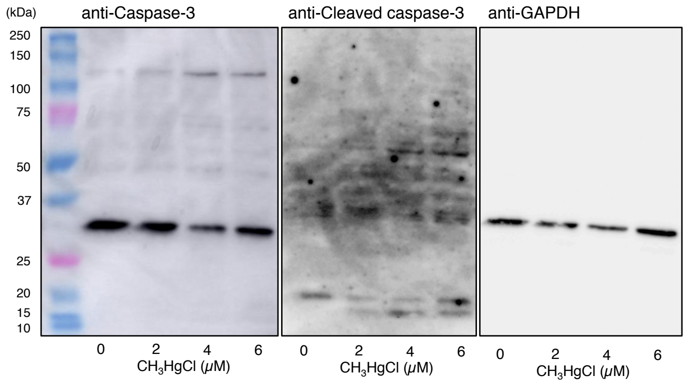

d **Figure 7k**

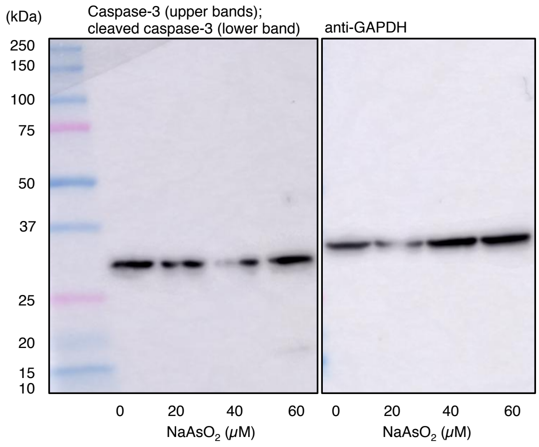

e **Figure 7n**

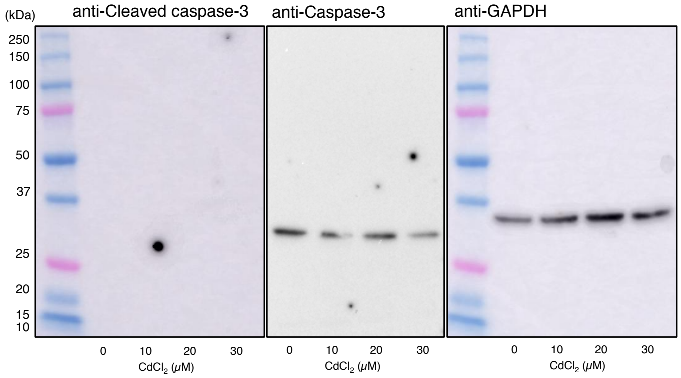

Supplementary Figure 4. Original images of cropped blots in supplementary figure 1.

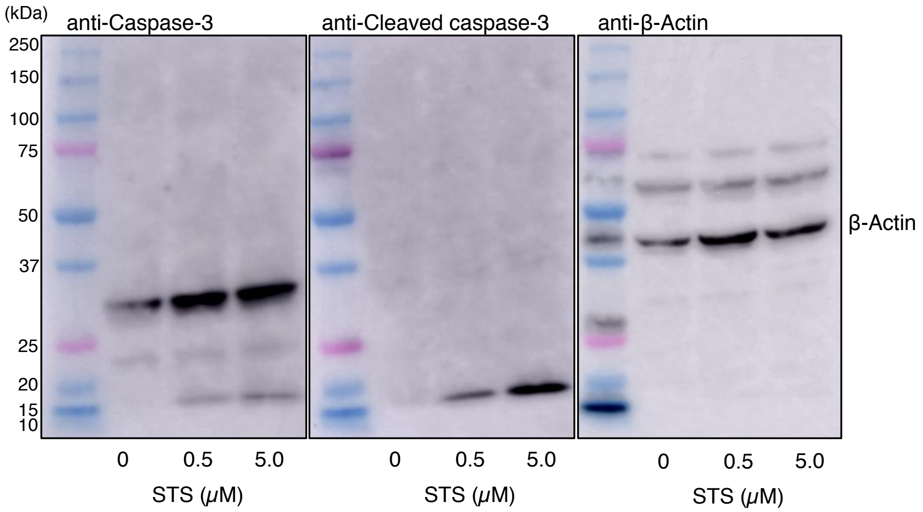

Supplement: Supplementary file 1 — Supplementary information [file 41598_2017_17494_MOESM1_ESM.pdf]
